# Killian-Jamieson Diverticulum: Management of a Rare Esophageal Diverticula

**DOI:** 10.7759/cureus.17820

**Published:** 2021-09-08

**Authors:** James Oh, Ashton Norris, Michael Artigue, Jessica Kruger

**Affiliations:** 1 General Surgery, University of North Texas Health Science Center-Texas College of Osteopathic Medicine/John Peter Smith Hospital, Fort Worth, USA; 2 General Surgery, Baylor University Medical Center, Dallas, USA

**Keywords:** killian-jamieson diverticulum, zenker diverticulum, esophageal diverticulum, surgical technique, clinical management, clinical approaches & management

## Abstract

Killian-Jamieson diverticulum (KJD) is a rare presentation of esophageal diverticulum. It is located beneath the cricopharyngeal muscle and arises laterally from the Killian-Jamieson space. The pathogenesis is postulated to be from increased intraluminal pressure. Most patients with KJD are typically asymptomatic; however, a common clinical presentation is dysphagia. Demographics of patients with KJD are typically elderly, in which the majority are female and over 50 years old. Due to less frequent diagnosis of KJD, there are a limited number of case studies compared to Zenker’s diverticulum, the more common presentation of esophageal diverticulum. In this case study, we discuss an atypical case presentation in a young, African-American female.

## Introduction

Killian-Jamieson diverticulum (KJD) is a rare presentation of esophageal diverticulum. While Zenker’s diverticulum (ZD) is more prevalent and found arising in Killian’s dehiscence above the cricopharyngeal muscle, KJD arises in the Killian-Jamieson space below the cricopharyngeal muscle. Furthermore, ZD extends posteriorly as it protrudes compared to KJD extending laterally. The demographics of KJD are similar to those of ZD in terms of elder age, usually over 50 years old, but differ with a higher prevalence in females. In the effort to augment the scant literature on a rare diagnosis, we report an atypical case of KJD in a symptomatic 39-year-old African American female, significantly younger than the commonly reported age. We discuss the workup and management of our patient with transcervical diverticulectomy and esophageal myomectomy. 

## Case presentation

**Figure 1:**
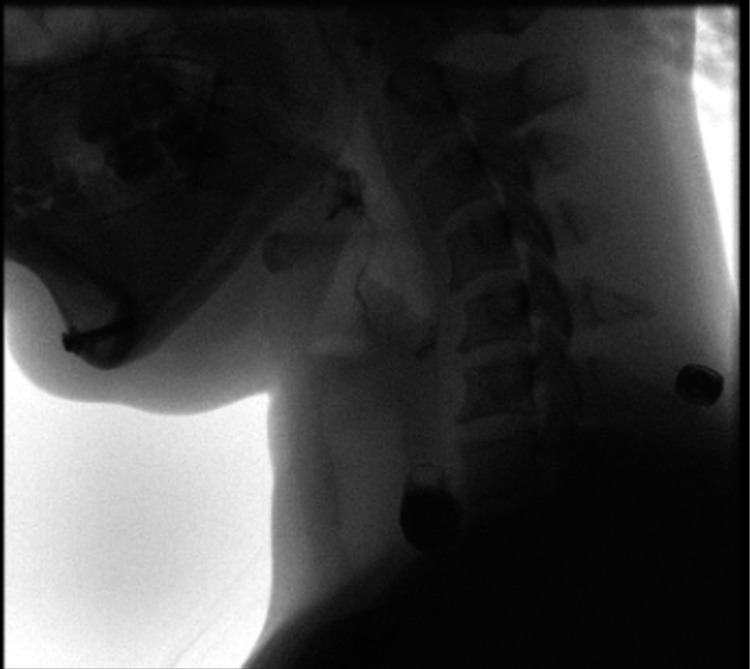
Video Fluoroscopy Swallow Study, Lateral View. Collection of contrast within the Killian-Jamieson diverticulum.

**Figure 2:**
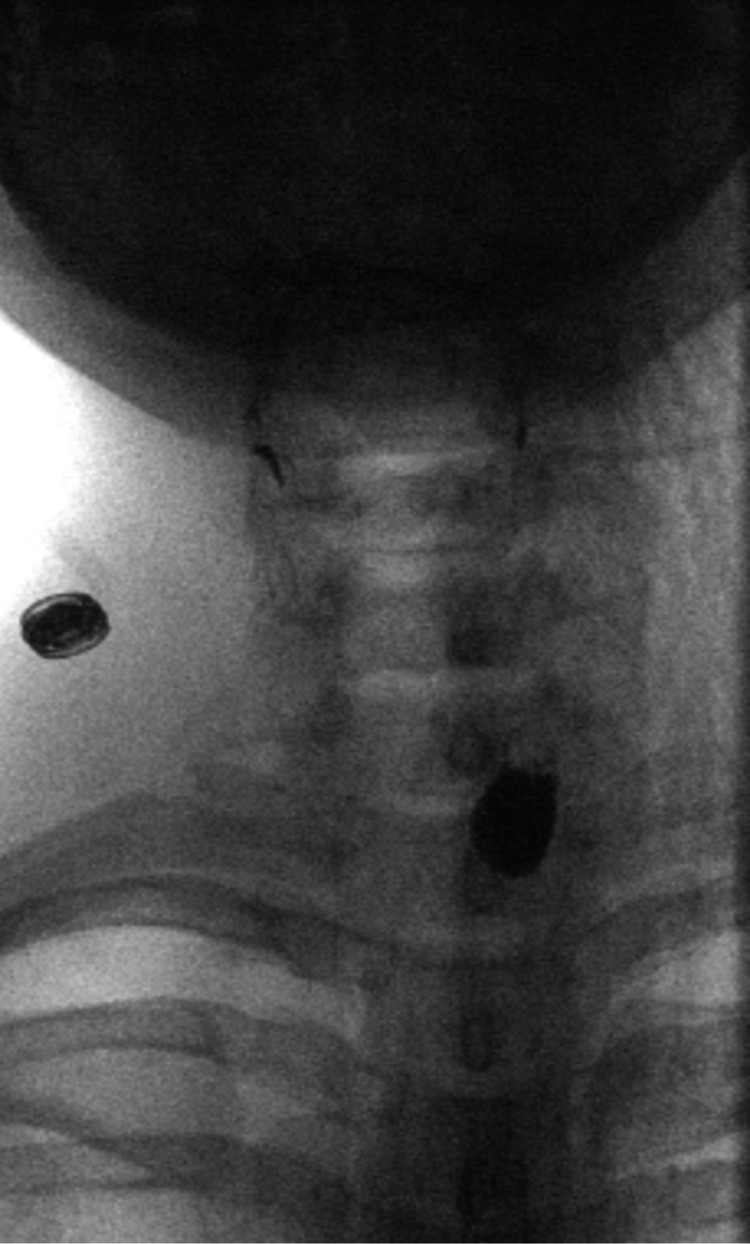
Video Fluoroscopy Swallow Study, Anterior View. Collection of contrast laterally, suggestive of Killian-Jamieson diverticulum.

A 39-year-old African American female with a past medical history of anxiety, asthma, gastroesophageal reflux disease, and *Helicobacter pylori* infection presented to the emergency department with dysphagia. For several months, the patient reported persistent episodes of “choking” and globus sensation with recurrent cough after eating solid food. The problem began with an episode of choking on steak. She denied problems with liquid intake, but the severity of her dysphagia led to anxiety and food aversion, causing her to lose an estimated 30 lbs. The patient endorsed an additional associated symptom of orthopnea, but denied chest pain and food regurgitation. Initial physical examination and lab workups were unremarkable. A subsequent Barium swallow pharyngoesophagography demonstrated a 1.0 cm x 1.9 cm left-sided, anterolateral esophageal diverticulum located beneath the cricopharyngeus muscle, consistent with KJD (Figures 1, 2). 

We reviewed the current literature to find the best approach in management for our patient. We discovered the treatment for symptomatic KJD is surgical; however, the approach is debated. The preferred method is transcervical diverticulectomy, with or without esophageal myotomy. The less commonly used method is endoscopic diverticulectomy due to concern for recurrent laryngeal nerve damage. Based on our findings, we planned for a transcervical diverticulectomy with esophageal myotomy. The risks and benefits were discussed with the patient, to which she consented.

**Figure 3:**
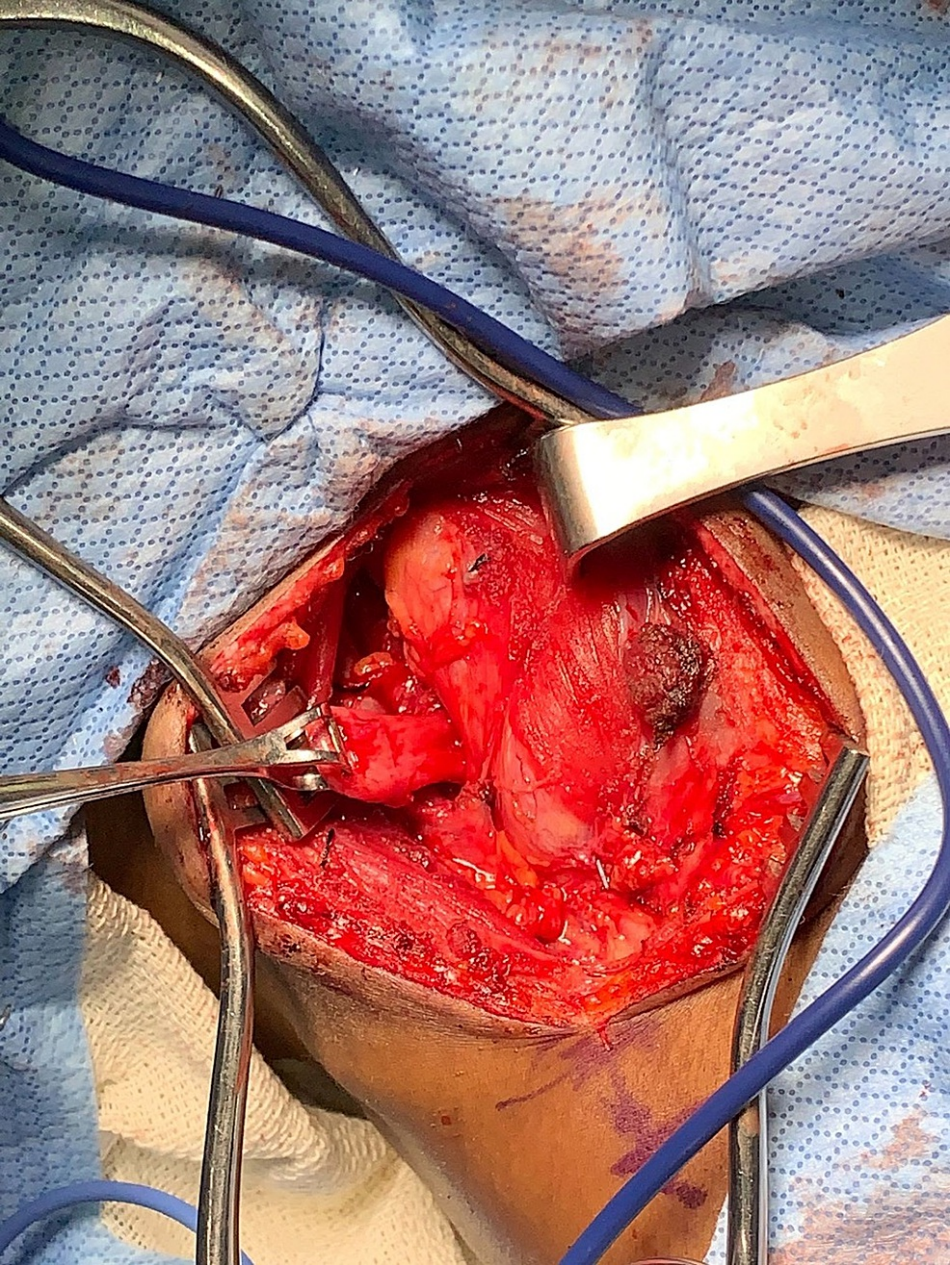
Killian-Jamieson Diverticulum. Surgical view.

A 15-blade scalpel was used to make the incision anterior to the sternocleidomastoid. The subcutaneous tissue and platysma were dissected. The sternocleidomastoid was mobilized off the underlying tissue and the omohyoid was then transected. The carotid sheath was identified and protected. The KJD was found and carefully cleared of surrounding attachments. The left recurrent laryngeal nerve (LRN) was identified anterior to the esophagus via Nims monitor and confirmed to be intact. A 1-cm myotomy of the cricopharyngeal muscle was undertaken cranial to the diverticulum. A 38 French Bougie was placed into the esophagus by anesthesia to delineate the KJD from normal esophageal anatomy and the diverticulum was transected using a 35-mm Echelon Flex Vascular Stapler. Hemostasis was achieved following transection and a 15 French Jackson-Pratt drain was placed in the wound and brought out lateral to the incision, secured with nylon suture. The platysma fascia was closed with interrupted vicryl sutures and the skin closed with running 4-0 monocryl and dermabond (Figure 3).

## Discussion

Named after physicians Gustav Killian and James Jamison, KJD was first described by Ekberg and Nylander in their 1983 paper as a lateral diverticulum from the pharyngeal-esophageal junction [1]. KJD is a rare presentation of esophageal diverticulum and is differentiated from the more common ZD based on its location. KJD protrudes from the Killian-Jamieson space, an area bounded inferiorly by the cricopharyngeus muscle and laterally by the longitudinal muscle of the esophagus [1]. This space is distinguished from ZD origination, Killian’s dehiscence (also known as Killian’s triangle), which is bordered superiorly by the inferior pharyngeal constrictor muscle and inferiorly by the cricopharyngeus [2]. The average size of KJD ranges between 2.8 and 3.8 cm [3]. In comparison, the ZD’s average size is 6.0 cm and typically twice the size of the average KJD [4]. There appears to be a size relationship to symptom presentation as patients with ZD are more likely to be symptomatic [5]. The demographics of KJD are similar to ZD patients, where the majority of patients are elderly, with an average age of 72 and 66 years, respectively [5]. The prevalence of ZD and KJD is 0.01%-0.11% and 0.025%, respectively, in the general population [6]. ZD is more commonly found in men [7] while KJD is more commonly found in women [8].

The pathogenesis of KJD remains unclear. Based on the literature review, several hypotheses have been suggested. Tang et al. postulated significant increase in intraluminal pressure secondary to functional outflow obstruction due to contraction of the circular esophageal muscle [9]. Per Bosivert et al., the etiology of ZD and KJD appears to share a common pathophysiology [10]. 

The vast majority of patients with KJD are asymptomatic. The general increased size of ZD in comparison to KJD likely results in less symptoms and reports. If patients do become symptomatic, history alone can be challenging to differentiate between KJD and ZD. Patients most commonly present with dysphagia in both ZD and KJD [11]. Other recurrent symptoms include globus sensation, cough, halitosis, aspiration, and neck pain [11].

Definitive diagnosis is typically clinched with imaging as barium esophagram demonstrates the location, approximate size, and the lateralization [12]. Other modalities include esophageal endoscopy, CT scan, and ultrasound; however, most clinicians rest their clinical decisions based on barium esophagram [12]. The decision to proceed with esophageal manometry and esophageal pH probe is controversial. Esophageal manometry has been used to decipher the etiology of ZD [13] and Migliore et al. recommend surgical intervention only after manometric evaluation [14]. However, other studies showed inconsistent upper esophageal sphincter pressures with varying timing of contraction [15,16] suggesting that the manometry is an unreliable tool for ZD diagnosis. 

For patients who are asymptomatic, expectant management is a reasonable option [17]. Multiple surgical options are available for those who develop symptoms, including esophagomyotomy with transcervical diverticulectomy [18]. This is the preferred approach due to concern for inadvertent impairment of the LRN given its proximity to the KJD. Endoscopic diverticulotomy is another option and has been a proven treatment for ZD because the LRN has been shown to be safely preserved throughout the procedure [18].

## Conclusions

Our patient did not meet the typical elderly demographics, demonstrating that a relatively young, healthy patient can develop KJD. Therefore, we recommend clinicians having KJD as one of their differentials for young patients presenting with dysphagia. The treatment of choice is transcervical diverticulectomy with or without esophageal myomectomy. Endoscopic diverticulectomy is an alternative treatment option but not the preferred approach due to potential injury of the recurrent laryngeal nerve.
